# A Comprehensive Phenotype of Non-motor Impairments and Distribution of Alpha-Synuclein Deposition in Parkinsonism-Induced Mice by a Combination Injection of MPTP and Probenecid

**DOI:** 10.3389/fnagi.2020.599045

**Published:** 2021-01-13

**Authors:** Na-Ra Han, Yu-Kang Kim, Sora Ahn, Tae-Yeon Hwang, Hyejung Lee, Hi-Joon Park

**Affiliations:** ^1^Integrative Parkinson’s Disease Research Group, Acupuncture and Meridian Science Research Center, Kyung Hee University, Seoul, South Korea; ^2^Department of Anatomy & Information Sciences, College of Korean Medicine, Kyung Hee University, Seoul, South Korea; ^3^Department of Meridian & Acupoints, College of Korean Medicine, Kyung Hee University, Seoul, South Korea

**Keywords:** Parkinson’s disease, non-motor symptoms, α-synuclein, 1-methyl-4-phenyl-1, 2, 3, 6-tetrahydropyridine and probenecid, behavior tests

## Abstract

Parkinson’s disease (PD) is characterized by non-motor symptoms as well as motor deficits. The non-motor symptoms rarely appear individually and occur simultaneously with motor deficits or independently. However, a comprehensive research on the non-motor symptoms using an experimental model of PD remains poorly understood. The aim of the current study is to establish a chronic mouse model of PD mimicking the comprehensive non-motor symptoms of human PD by injection of 1-methyl-4-phenyl-1,2,3,6-tetrahydropyridine (MPTP) and probenecid (MPTP/p). The non-motor and motor symptoms were evaluated by performing buried food, short-term olfactory memory, hot plate, open field, tail suspension, Y maze, novel object recognition, bead expulsion, one-h stool collection, rotarod, rearing, catalepsy, and akinesia tests after 10 injections of MPTP/p into mice. The expression levels of α-synuclein, glial fibrillary acidic protein (GFAP), tyrosine hydroxylase (TH) or DJ-1 were analyzed by Western blotting or immunostaining. MPTP/p-treated mice achieved to reproduce the key features of non-motor symptoms including olfactory deficit, thermal hyperalgesia, anxiety, depression, cognitive decline, and gastrointestinal dysfunction in addition to motor deficits. The MPTP/p-treated mice also showed the high levels of α-synuclein and low levels of TH and DJ-1 in striatum, substantia nigra, olfactory bulb, hippocampus, amygdala, prefrontal cortex, locus coeruleus, or colon. In addition, the expression levels of phosphorylated-α-synuclein and GFAP were elevated in the striatum and substantia nigra in the MPTP/p-treated mice. Taken together, our study clarifies that the chronic MPTP/p-treated mice have a variety of non-motor dysfunctions as well as motor abnormalities by α-synuclein overexpression and dopaminergic depletion. Therefore, the study of comprehensive phenotypes of non-motor symptoms in one PD model would advance in-depth understandings of neuropathological alternations and contribute to future strategies for PD treatment.

## Introduction

Parkinson’s disease (PD) is currently regarded as the most common neurodegenerative disorder following Alzheimer’s disease, that affects over 5 million people worldwide and 1–3% of people over 50 years of age (Meissner et al., 2011; Lashuel et al., 2013). PD is mainly characterized by motor deficits including tremor or rigidity (Dauer and Przedborski, 2003). These motor deficits result from severe loss of dopaminergic nigrostriatal neurons (Maiti et al., 2017). Recently, however, a wide range of non-motor symptoms such as olfactory deficit or cognitive decline is known to be strongly associated with PD pathological processes and significantly affect the quality of life of PD patients (Schapira et al., 2017; Monastero et al., 2018; Liu et al., 2020).

α-synuclein is a major pathological protein that underlies PD pathogenesis (Butkovich et al., 2018). The widespread aggregation of α-synuclein is a neuropathological hallmark of PD (Dehay et al., 2015). The progressive and abnormal accumulation of α-synuclein in various brains regions leads to neurodegeneration and impairment of neurotransmitter release (Lashuel et al., 2013). In addition, the overexpression of α-synuclein in striatum, substantia nigra, olfactory bulb, hippocampus, amygdala, prefrontal cortex, locus coeruleus, and colon are involved in non-motor features such as anxiety, depression or cognitive decline (Jellinger, 2011; Shannon et al., 2012; Flores-Cuadrado et al., 2016; Schapira et al., 2017; Butkovich et al., 2018; Henrich et al., 2018; Stoyka et al., 2020). The high level of phosphorylated (p)-α-synuclein is a pathological deposition of α-synuclein in correlation with the behavioral phenotypes of PD (Canerina-Amaro et al., 2019). One of phosphorylated forms of α-synuclein, pS129, is significantly correlated with PD severity (Wang et al., 2012; Stewart et al., 2015).

Animal models have been developed to identify pathological mechanisms of PD using several neurotoxins such as 1-methyl-4-phenyl-1,2,3,6-tetrahydropyridine (MPTP) as reviewed by Zeng et al. (2018). Among the neurotoxins, MPTP crossed blood–brain barrier after systemic administration and exhibited the notable degeneration of nigrostriatal dopaminergic neurons in a PD model (Zeng et al., 2018). Thus, an MPTP-intoxicated mouse model has been commonly used as an animal PD model (Chan et al., 2007; Jackson-Lewis and Przedborski, 2007). However, the dopamine loss was rapid and not progressive, so it was often difficult to determine the motor disability of PD in most MPTP models (Meredith et al., 2008). Motor deficits appeared in several acute or subacute MPTP mouse models (Li et al., 2016; Sim et al., 2017), but other MPTP models hardly mimicked the motor deficits of human PD (Rousselet et al., 2003; Luchtman et al., 2009; Zhang et al., 2017). Chan et al. (2007) suggested that the chronic injection of both MPTP and probenecid (MPTP/p) induces pathological hallmarks of PD with motor deficits, making it an appropriate excellent choice for pathogenic researches on PD.

The most of PD patients have one or more non-motor symptoms (Shalash et al., 2018). There was a clinically significant correlation among non-motor signs (Bugalho et al., 2016). However, studies using a PD mouse model have been limited to a specific non-motor symptom or the lack of a comprehensive approach to non-motor symptoms (Laloux et al., 2008; Fox et al., 2010; Park et al., 2015; Ellett et al., 2016). The mutual neuropathological study of non-motor symptoms and establishment of an experimental model representing the comprehensive non-motor symptoms of human PD are needed to clarify the complexity of PD. Thus, the aim of the current study is to establish an experimental model of PD with the spectrum of non-motor symptoms in addition to motor impairments caused by the chronic injection of MPTP/p, which mimics characteristics of human PD.

## Materials and Methods

### Animals

Eight-week-old male C57BL/6 mice (24–26 g, Central Laboratories Animal Inc., Seoul, South Korea) were maintained under a standard laboratory condition (12 h light–dark cycle, 55 ± 10% relative humidity and 22 ± 1°C). We used male mice for establishing a PD mouse model with reference to the previous reports (Lei et al., 2012; Brichta et al., 2015; Maatouk et al., 2018). Mice were given *ad libitum* access to water and feed. Experimental procedures were performed with reference to the National Institutes of Health Guide for the Care and Use of Laboratory Animals. The procedures were also approved by the Dongguk University Animal Care Committee (IACUC-2017-023-1).

### Experimental Procedures

**FIGURE 1:**
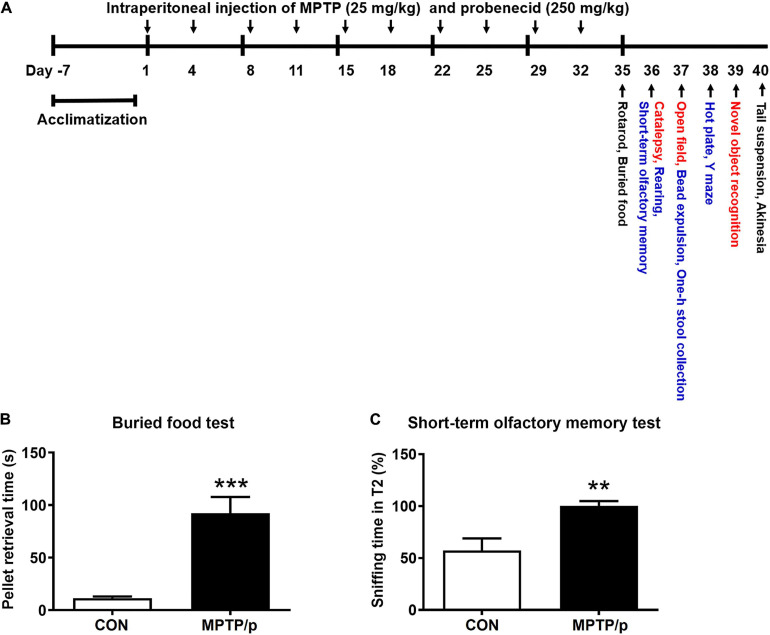
The chronic injection of MPTP/p causes olfactory deficit in mice. **(A)** Scheme of the experimental procedure. MPTP/p was intraperitoneally administered twice a week for 5 weeks, a total of 10 times. On day 35, the mice were first subjected to the rotarod test and buried food test (black; *n* = 14/group). The next day, mice were randomly divided into two kinds of tests (red; *n* = 7/group and blue; *n* = 7/group). On day 40, all mice were combined to perform the tail suspension test and akinesia test (black; *n* = 14/group). **(B)** The time (s) required to find the buried food pellet in the buried food test (*n* = 14/group) on day 35 of exposure to MPTP/p described in Materials and Methods section. **(C)** Time (%) to sniff the odor at T2 compared to the total time to sniff at both T1and T2 in the short-term olfactory memory test (*n* = 7/group) on day 36 of exposure to MPTP/p described in Materials and Methods section. Graphed data are mean ± SEM. Significant differences from values of control mice by unpaired Student’s *t*-test are indicated as **P < 0.01; ***P < 0.001. CON, control; MPTP/p, MPTP and probenecid; T1, trial 1; T2, trial 2.

MPTP (Cat# 16377, Cayman Chemical, Ann Arbor, MI, United States) was dissolved in saline and prepared at a concentration of 25 mg/kg according to a report of Shao et al. (2019). Probenecid (Cat# 14981, Cayman Chemical) was dissolved in dimethyl sulfoxide and prepared at a concentration of 250 mg/kg according to a report of Shao et al. (2019). Experimental procedures (Figure 1A) were as follows. Mice were randomly grouped into two groups (MPTP/p group and control group) after a week of acclimatization. MPTP/p was intraperitoneally administered twice per week for 5 weeks (Chan et al., 2007; Luchtman et al., 2009; Shao et al., 2019). Because probenecid as an adjuvant maintains the effectiveness of neurotoxins of chronic MPTP by reducing the renal excretion of MPTP and its metabolites, probenecid was administered 30 min before the MPTP injection (Meredith et al., 2008; Choi et al., 2018). Control mice were injected with saline and dimethyl sulfoxide twice a week for 5 weeks. Three days after the last injection, on day 35, the non-motor symptoms and motor dysfunctions were evaluated using various behavior tests. All animals were then sacrificed for further study.

### Behavior Tests

The behavior tests are listed in Table 1. All behavior tests were conducted by blind observers between 9: 00 and 18: 00. On day 35, the mice were first subjected to the rotarod test and buried food test (Figure 1A, black; *n* = 14/group). In order to minimize the stress of the experimental animals, mice were randomly divided into two kinds of tests (Figure 1A, red; *n* = 7/group and blue; *n* = 7/group) the next day. On day 40, all mice were combined to perform the tail suspension test and akinesia test (black; *n* = 14/group).

**TABLE 1:** Behavior tests.

**Impairments of Parkinson’s disease**	**Behavior tests**				**References**
Olfactory deficit	Buried food	Short-term olfactory memory			Yang and Crawley, 2009; Petit et al., 2013
Thermal hyperalgesia	Hot plate				Challis et al., 2020
Anxiety and depression	Open field	Tail suspension			Seibenhener and Wooten, 2015; Badr et al., 2020
Cognitive decline	Y maze	Novel object recognition			Fernandez et al., 2017; Yan et al., 2017
Gastrointestinal dysfunction	Bead expulsion	One-h stool collection			Li et al., 2006; Natale et al., 2010
Motor dysfunction	Rotarod	Rearing	Catalepsy	Akinesia	Lu et al., 2014

#### Buried Food Test

To assess the olfactory deficit, we performed the buried food test as previously described with a few modifications (Yang and Crawley, 2009). The buried food test measures how quickly food-restricted mice find hidden food. The mice that are unable to find the food within a 15 min period are assumed to have olfactory dysfunctions. Mice usually find the hidden piece of food in a few min (Yang and Crawley, 2009). For the buried food test, a clean mouse cage was filled with clean litter 3–4 cm deep. Mice were subjected to fasting without water and feed 24 h before the test. The mouse was placed in any corner of the cage. The pieces of cheese were buried in a different location. The litter was changed between mice. The time until the mouse found the hidden piece of cheese (when the mouse touched the pellet) was measured.

#### Short-Term Olfactory Memory Test

We performed the short-term olfactory memory test as another test to evaluate the olfactory deficit as previously described (Petit et al., 2013). It is expected that mice would spend less time sniffing in the second trial (T2) if they remember the odor in the first trial (T1) (Petit et al., 2013). We exposed a mouse to the lemon juice odor at two 5 min-trials separated by 120 s inter-trial intervals. The sniffing time was defined as head up sniffing within 1 cm of the odor source. It was analyzed by video recording and expressed as a percentage of time to sniff the odor at T2 compared to the total time to sniff at both T1 and T2.

#### Hot Plate Test

The hot plate test was performed to evaluate the thermal hyperalgesia as previously described (Challis et al., 2020). The latency time is measured after the first occurrence of the following endpoint nocifensive behaviors: flinching, hind paw licking or jumping. Since the forefoot is used for grooming and exploration, and not consistently in contact with the metal surface, licking, or withdrawal of the hind paw is considered a more reliable indicator of nociception (Deuis et al., 2017). A mouse was habituated to an unheated hot plate (Bioseb – *In Vivo* Research Instruments, Pinellas Park, FL, United States; Model: BIO-CHP) for 5 min. The latency time taken to elicit the nocifensive behaviors was measured after the mouse was placed on the metal surface maintained at a constant temperature 52.5°C. The cutoff time was set to 40 s to prevent a paw thermal injury.

#### Open Field Test

To assess the anxiety-related behaviors, we performed the open field test as previously described (Park et al., 2015). Thigmotaxis or wall-hugging behavior is associated with anxiety-related behaviors to avoid wide open areas and regarded as the starting point for anxiety-related tests (Seibenhener and Wooten, 2015). The center zone size was designated as 20 cm × 20 cm (Bailey and Crawley, 2009). A mouse was placed in an open top and square box (40 cm × 40 cm × 24 cm) in a quiet room and able to freely move for 5 min under bright illumination. The box was thoroughly wiped with 10% ethanol between each test to remove any residue. The number of entries into center zone, total movement distance, and central movement distance were analyzed with Smart 3.0 software (Panlab Harvard Apparatus, Barcelona, Spain). Finally, the anxiety-like behavior was examined as a ratio of central/total distance.

#### Tail Suspension Test

To assess the depression-like behaviors, we performed the tail suspension test as previously described (Badr et al., 2020). The immobility is a behavioral characteristic associated with depression (Cryan et al., 2005). A mouse was suspended 50 cm above the ground using plastic boxes (50 cm × 50 cm × 50 cm) and adhesive tape about 1 cm from the tip of tail. The immobility time was considered when the mouse hung completely motionless. We recorded the immobility time during 6 min.

#### Y Maze Test

To evaluate the cognitive decline, we performed the Y maze test to analyze immediate spatial working memory as a form of short-term memory as previously described (Yan et al., 2017). Mice generally prefer a new arm rather than returning to the previously visited location in the maze. Spontaneous alternations of mice are considered to enter the other arms in succession, i.e., ABC, BAC or CBA, but not CAC (CAC means repetitive), which means that mice have immediate spatial working memory (Yan et al., 2017). The Y-maze consisted of three white acrylic arms (30 cm × 5 cm × 15 cm) with a common neutral area (triangular neutral zone) in the center (5 cm × 5 cm × 15 cm). A mouse was placed in an arbitrary arm and able to freely explore through the maze for 5 min. The Y maze arms were wiped with 10% ethanol between tests. The alternation was expressed as a ratio of actual alternations to possible alternations (total arm entries – 2). The equation is as follows: alternation (%) = [number of alternations/(total arm entries – 2)] × 100.

#### Novel Object Recognition Test

We performed the novel object recognition test as another test to evaluate the cognitive decline as previously described with a few modifications (Fernandez et al., 2017). The novel object recognition test is based on the spontaneous characteristic of mice that spend more time exploring novel objects than familiar objects. The choice of exploring novel objects implies the use of cognitive memory and learning (Antunes and Biala, 2012). The novel object recognition test was composed of three sessions (habituation, training and testing). Each session was conducted once a day for three consecutive days. The first day consisted of a 30-min habituation session in a white arena (40 cm × 40 cm × 24 cm). On the second day of training session, the mouse was able to freely move for 10 min after two identical objects were centered. On the third day of testing session, one object (familiar object) was replaced with a novel object. The novel object was made of the same material with different shapes and colors. The mouse was allowed to explore for 5 min. Through video recording, the exploration time for each object, the number of entries into the novel object zone (within a 3 cm radius of the object) and discrimination index were automatically analyzed by Smart 3.0 software (Panlab Harvard Apparatus). The exploration time for the familiar or novel object during the testing session was measured only when the nose pointed toward the objects at a distance of no more than 2 cm and/or touched or sniffed the object. Memory was determined by the discrimination index as shown by the following equation: [Discrimination index = (novel object exploration time/total exploration time) – (familiar object exploration time/total exploration time) × 100].

#### Bead Expulsion Test

We performed the bead expulsion test to evaluate the gastrointestinal dysfunction as previously described (Natale et al., 2010). Mice were subjected to fasting without water and feed 24 h before the test. Mice were temporarily anesthetized with isoflurane (Ifran liquid for inhalation, HANA Pharm Co., Seoul, South Korea). The glass 3-mm bead (Cat# 4901003, Paul Marienfeld GmbH & Co. KG, Lauda-Königshofen, Germany) was inserted 2 cm into distal colon. After bead insertion, the mouse was individually placed in a clean plastic cage. Bead expulsion time was measured within 100 min as a time limit. We measured the bead expulsion latency (the time required for the bead expulsion) and expressed the time as a percentage compared to that of control mice.

#### One-h Stool Collection Test

We performed the one-h stool collection test as another test to evaluate the gastrointestinal dysfunction as previously described (Li et al., 2006). Each mouse was placed in a separate clean plastic cage and observed during the one-h collection period. Stools were collected immediately after being expelled and placed in sealed laboratory tubes. The wet weights of the stools in tube were measured. The stools were then dried in an oven at 65°C. The stool water contents were calculated from differences between the wet and dry stool weights.

#### Rotarod Test

The rotarod (MED associates Inc., Albans, VT, United States) test was performed to assess the motor dysfunction as previously described (Park et al., 2017; Parker et al., 2018). Briefly, we trained mice for 3–4 days on an accelerated rotarod so that the mice remained on the rotarod. The test was performed three times with the rod accelerating linearly from 3.5 to 35 rpm within 2 min. The test session consisted of 480 s intervals. The time on rod of the mice was measured.

#### Rearing Test

The rearing test was performed to evaluate the motor impairments as previously described (Park et al., 2017). A mouse was placed in a plastic cylinder (12 cm diameter × 20 cm height) under dim light conditions and adapted for 1 min before the experiment. The number of times the mouse forepaws touched the cylinder wall was counted for 3 min.

#### Catalepsy Test

The catalepsy test was conducted to assess the ability to correct an abnormally imposed body position (Lu et al., 2014) as previously described with a few modifications (Bardin et al., 2006). The two forelimbs of mouse were placed on a horizontal raised metal bar (1 cm diameter, 5 cm above the table) and their hindlimbs were placed on the floor of the apparatus. The time (latency time) during which both forelimbs remained on the bar was recorded before removing both forelimbs from the bar. The test was repeated three times (inter-trial interval: 1 min) and cutoff time was set to 180 s.

#### Akinesia Test

We conducted the akinesia test to assess the latency time to move all four limbs as previously described with a few modifications (Paul et al., 2017). A mouse was placed on a high and flat surface. A mouse was initially acclimatized for 5 min on the surface (40 cm × 40 cm × 24 cm) before the experiment. The time taken to move all the four limbs was measured. It was repeated 3 times and terminated if the latency exceeded 180 s.

### Immunohistochemistry and Quantitative Analysis

Mice were perfused with ice-cold paraformaldehyde (4%) in phosphate buffer (0.2 M). Brains were randomly removed from each group and post-fixed overnight in the fixative. After cryoprotection in sucrose/phosphate buffer (30%), the serial coronal sections of frozen brains were cut to 40 μm by a freezing microtome (Cat# CM1850, Leica, Nussloch, Germany). Free-floating sections were blocked with bovine serum albumin (1%)/phosphate buffer plus Triton X-100 (0.2%) and incubated with an anti-TH antibody (Santa Cruz Biotechnology, Santa Cruz, CA, United States) overnight at room temperature, followed by incubation with biotinylated secondary antibodies (Vector Laboratories, Inc., Burlingame, CA, United States) and ABC reagents (Vectastain Elite ABC kit, Vector Laboratories, Inc.). The sections developed with diaminobenzidine (Sigma-Aldrich Co., St. Louis, MO, United States) were dehydrated and coverslipped. A bright-field microscope (Cat# BX51, Olympus Japan Co., Tokyo, Japan) was used for observing images. The optical density of TH in striatum was analyzed using ImageJ software (National Institute of Health, Bethesda, MD, United States). The number of TH + neurons in substantia nigra was stereologically analyzed with an unbiased optical fractionator method that is not affected by either the volume of substantia nigra or size of the counted neurons. We used a computer-assisted image analysis system consisting of an Axiophot photomicroscope (Carl Zeiss Vision International GmbH, Aalen, Germany) equipped with a computer controlled motorized stage (Ludl Electronics, Hawthorne, NY, United States), a Hitachi HV C20 camera and Stereo Investigator software (MicroBright-Field, Williston, VT, United States). The total number of TH + neurons was calculated as previously described (Mandir et al., 1999).

### Western Blot Analysis

Brains were randomly removed from each group. Tissues from striatum, substantia nigra, olfactory bulb, hippocampus, amygdala, prefrontal cortex, locus coeruleus, and mid part of colon were homogenized in CyQUANT^®^ cell lysis buffer (Invitrogen, Carlsbad, CA, United States). After the homogenates were centrifuged, the protein concentration was measured with a BCA protein assay (Sigma-Aldrich Co.). The equal protein amounts of each sample were combined with SDS-polyacrylamide gel electrophoresis (SDS-PAGE) loading buffer (Biomedic Co., Bucheon, South Korea), heated at 95°C for 10 min, separated by SDS-PAGE gels (10%) and transferred onto nitrocellulose membranes. The membranes blocked with skimmed milk (5%) were incubated with the following primary antibodies: α-synuclein (1:500 dilution, Cell Signaling Technology, Danvers, MA, United States), p-α-synuclein (pS129, 1:200 dilution, Abcam, Cambridge, United Kingdom), glial fibrillary acidic protein (GFAP, astroglial marker, 1:500 dilution, Invitrogen), ionized calcium-binding adapter molecule-1 (Iba-1, microglial marker, 1:500 dilution, Sigma-Aldrich Co.), TH (1:1,000 dilution, Santa Cruz Biotechnology), DJ-1 (1:1,000 dilution, Cell Signaling Technology) and β-actin (1:4,000 dilution, Sigma-Aldrich Co.), followed by horseradish peroxidase-conjugated secondary antibodies (1:10,000 dilution, Pierce Biotechnology, Rockford, IL, United States). Bands were visualized by an enhanced chemiluminescence system (West Pico, Pierce Biotechnology). Densitometry analysis was performed using ImageJ software (National Institute of Health).

### Statistical Analysis

All data were analyzed by the unpaired Student’s *t*-test depending on the homogeneity of variance (IBM SPSS statistics 23, IBM Corp., Armonk, NY, United States). *P* values lower than 0.05 were considered to be significant. Asterisks signs indicate statistical significance between control mice versus MPTP/p-treated mice: ^∗^*P* < 0.05; ^∗∗^*P* < 0.01; ^∗∗∗^*P* < 0.001. Graphed data are mean ± standard error of the mean (SEM).

## Results

### The Chronic Injection of MPTP/p Causes Olfactory Deficit in Mice

The most widely studied non-motor feature is olfaction, which has been found to be abnormal in more than 90% of PD patients (Doty et al., 1988). Thus, it was first investigated whether olfactory deficit would be caused in the MPTP/p-induced PD mouse model by performing the buried food test and short-term olfactory memory test. The major parameter of the buried food test is the time required to find the buried food pellet (Yang and Crawley, 2009). The MPTP/p-treated mice significantly increased the retrieval time, showing difficulties in detecting the buried food (Figure 1B, *P* < 0.0001). Next, we conducted the short-term olfactory memory test as another olfactory deficit test. As shown in Figure 1C, control mice spent less time sniffing the novel odor on the second exposure, behaving as if they remembered having been exposed to the odor before (Petit et al., 2013). However, the MPTP/p-treated mice spent more time sniffing the odor, behaving as if they were unable to remember the odor (Figure 1C, *P* = 0.0052).

### The Chronic Injection of MPTP/p Causes Thermal Hyperalgesia in Mice

**FIGURE 2:**
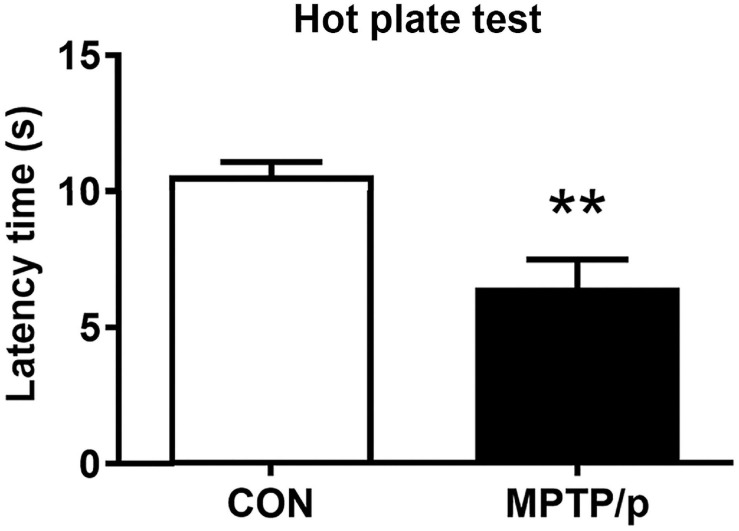
The chronic injection of MPTP/p causes thermal hyperalgesia in mice. MPTP/p was intraperitoneally administered twice a week for 5 weeks, a total of 10 times. The latency time (s) taken to elicit nocifensive behaviors (flinching, hind paw licking, or jumping) in the hot plate test (*n* = 7/group) on day 38 of exposure to MPTP/p described in Materials and Methods section. Values are mean ± SEM. Graphed data are mean ± SEM. Significant differences from values of control mice by unpaired Student’s *t*-test are indicated as **P < 0.01. CON, control; MPTP/p, MPTP and probenecid.

PD patients had hyperalgesia compared to normal controls (Sung et al., 2018a,b). Clinically, hyperalgesia plays an important role in the development of chronic pain (Sung et al., 2018a,b). Thus, we performed the hot plate test for evaluation of nociception in the MPTP/p-treated mice. As shown in Figure 2, the MPTP/p-treated mice significantly decreased the latency time, showing the onset of heat sensitivity was significantly advanced compared to control mice when the paws were exposed to heat (Figure 2, *P* = 0.0037).

### The Chronic Injection of MPTP/p Causes Neuropsychiatric Features in Mice

**FIGURE 3:**
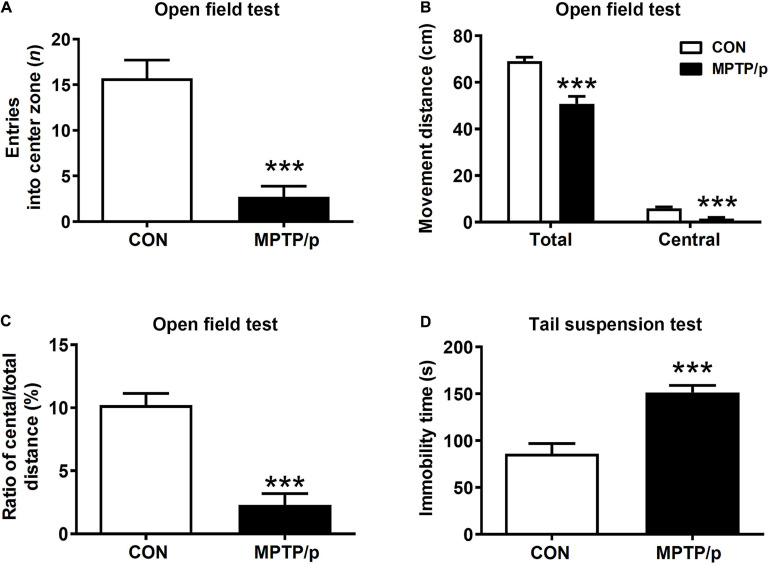
The chronic injection of MPTP/p causes neuropsychiatric features in mice. MPTP/p was intraperitoneally administered twice a week for 5 weeks, a total of 10 times. **(A)** Entries (*n*) into center zone, **(B)** total movement distance (cm) and central movement distance (cm) of their respective tracks and **(C)** ratio (%) of central/total distance in the open field test (*n* = 7/group) on day 37 of exposure to MPTP/p described in Materials and Methods section. **(D)** Immobility time (s) of the tail suspension test (*n* = 14/group) on day 40 of exposure to MPTP/p described in Materials and methods section. Graphed data are mean ± SEM. Significant differences from values of control mice by unpaired Student’s *t*-test are indicated as ***P < 0.001. CON, control; MPTP/p, MPTP and probenecid.

Anxiety affected approximately 60% of PD patients and was commonly accompanied by depression (Schapira et al., 2017). Thus, we performed the open field test and tail suspension test for evaluation of neuropsychiatric features in the MPTP/p-treated mice. The open field test is most commonly used to measure anxiety-related behaviors as well as ambulatory behaviors after MPTP intoxication (Park et al., 2015; Seibenhener and Wooten, 2015). The MPTP/p-treated mice significantly reduced the number of entries into center zone compared to control mice, suggesting a change in basal anxiety levels of the MPTP/p-treated mice (Figure 3A, *P* < 0.0001). The total movement distance (*P* = 0.0003), distance traveled in the center (central movement distance, *P* < 0.0001) and the ratio of central/total distance (*P* < 0.0001) were significantly reduced in the MPTP/p-treated mice (Figures 3B,C). In addition, the tail suspension test presented the depressive-like behaviors in the MPTP/p-treated mice with a significant increase in immobility time (Figure 3D, *P* < 0.0001).

### The Chronic Injection of MPTP/p Causes Cognitive Decline in Mice

**FIGURE 4:**
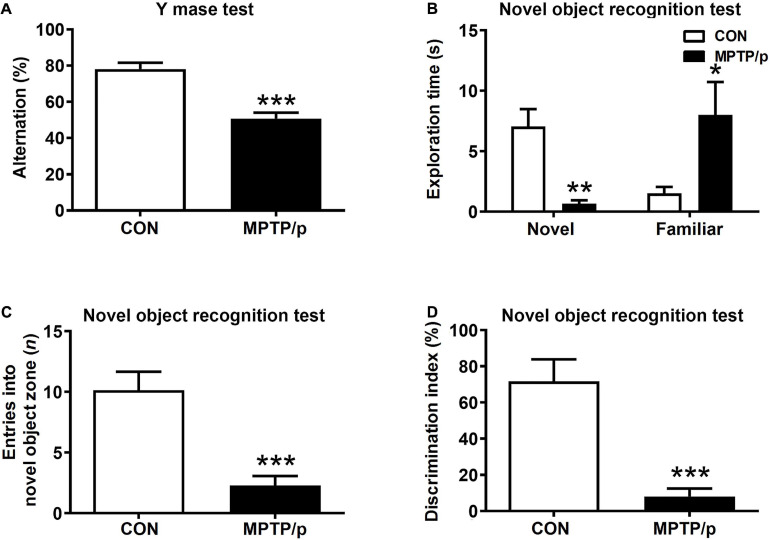
The chronic injection of MPTP/p causes cognitive decline in mice. MPTP/p was intraperitoneally administered twice a week for 5 weeks, a total of 10 times. **(A)** The percentage of alternation behaviors [number of alternations/(total arm entries – 2) × 100] in the Y maze test (*n* = 7/group) on day 38 of exposure to MPTP/p described in Materials and methods section. **(B)** Time (s) exploring the novel object and familiar object, **(C)** entries (*n*) into the novel object zone and **(D)** discrimination index (%) defined by equation [(novel object exploration time/total exploration time) – (familiar object exploration time/total exploration time) × 100] in the novel object recognition test on day 39 of exposure to MPTP/p described in Materials and Methods section (*n* = 7/group). Graphed data are mean ± SEM. Significant differences from values of control mice by unpaired Student’s *t*-test are indicated as *P < 0.05; **P < 0.01; ***P < 0.001. CON, control; MPTP/p, MPTP and probenecid.

People at risk of developing PD had cognitive decline and problems with working memory (Schapira et al., 2017). Thus, we explored whether the MPTP/p-treated mice would be defective in memory and learning by conducting the Y maze test and novel object recognition test. There was a significant difference in the spontaneous alternation between two groups of the Y maze test. The MPTP/p-treated mice significantly decreased the alternation behaviors (Figure 4A, *P* < 0.0001). In the novel object recognition test, the MPTP/p-treated mice also showed a significant deficit in the novel object recognition memory. The MPTP/p-treated mice spent significantly less time exploring the novel object (Figure 4B, *P* = 0.0082) and significantly more exploring the familiar object compared to control mice (Figure 4B, *P* = 0.0373). The number of entries into the novel object zone revealed that control mice preferentially approached to the novel object zone, but the MPTP/p-treated mice showed a striking decrease in the number of entries into the novel object zone (Figure 4C, *P* = 0.0006). The discrimination index also indicated that control mice exhibited a significant preference for exploring the novel object, but the MPTP/p-treated mice failed to show this preference (Figure 4D, *P* = 0.0003).

### The Chronic Injection of MPTP/p Causes Gastrointestinal Dysfunction in Mice

**FIGURE 5:**
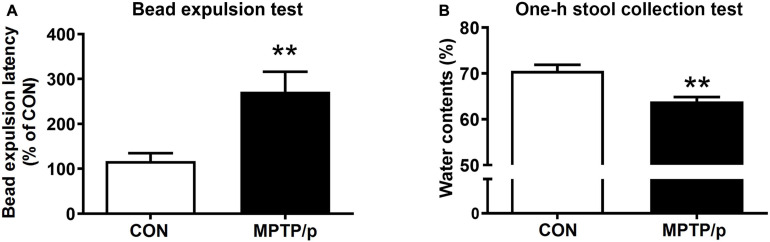
The chronic injection of MPTP/p causes gastrointestinal dysfunction in mice. MPTP/p was intraperitoneally administered twice a week for 5 weeks, a total of 10 times. **(A)** The bead expulsion latency (%) in the bead expulsion test (*n* = 7/group) on day 37 of exposure to MPTP/p described in Materials and Methods section. **(B)** Stool water contents (%) in the one-h stool collection test (*n* = 7/group) on day 37 of exposure to MPTP/p described in Materials and Methods section. Graphed data are mean ± SEM. Significant differences from values of control mice by unpaired Student’s *t*-test are indicated as **P < 0.01. CON, control; MPTP/p, MPTP and probenecid.

The gastrointestinal dysfunction is one of the most common non-motor impairments observed in PD, which often precedes the development of motor symptoms (O’Sullivan et al., 2008). Thus, we investigated whether the MPTP/p-treated mice would have gastrointestinal disturbances by performing the bead expulsion test and one-h stool collection test. The bead expulsion test, the MPTP/p-treated mice significantly increased the latency time to expel the bead compared to control mice, indicating a delay of enteric motility (Figure 5A, *P* = 0.0071). The one-h stool collection test also showed that water contents were significantly decreased by MPTP/p injection, indicating the MPTP/p-treated mice have constipation (Figure 5B, *P* = 0.0014).

### The Chronic Injection of MPTP/p Disrupts Motor Function in Mice

Additionally, we identified motor dysfunction as a major symptom of PD in the MPTP/p-treated mice by performing the rotarod test, rearing test, catalepsy test, and akinesia test. As expected, the MPTP/p-treated mice exhibited a significant decrease in the time on rod in the rotarod test, indicating the impaired movement function of the MPTP/p-treated mice (Supplementary Figure 1A, *P* < 0.0001). The MPTP/p-treated mice significantly reduced the number of touches on cylinder wall in the rearing test (Supplementary Figure 1B, *P* < 0.0001). The catalepsy test (Supplementary Figure 1C, *P* = 0.0010) and akinesia test (Supplementary Figure 1D, *P* < 0.0001) also showed that the latency times to start movement were significantly increased in the MPTP/p-treated mice, indicating PD-like abnormal behavior phenotypes of the MPTP/p-treated mice.

### The Chronic Injection of MPTP/p Leads to Abnormal Accumulation of α-Synuclein in Mice

**FIGURE 6:**
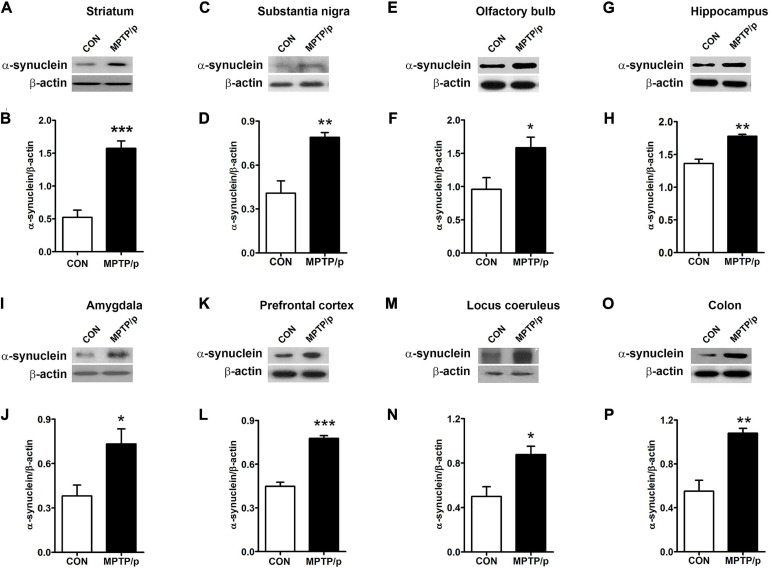
The chronic injection of MPTP/p leads to abnormal accumulation of α-synuclein in mice. Representative images of Western blotting analysis of α-synuclein in panels **(A)** striatum, **(C)** substantia nigra, **(E)** olfactory bulb, **(G)** hippocampus, **(I)** amygdala, **(K)** prefrontal cortex, **(M)** locus coeruleus, and **(O)** colon (*n* = 4/group). The other three Western blot images are shown in Supplementary Figure 2. **(B,D,F,H,J,L,N,P)** Quantification of normalized α-synuclein levels in each region. α-synuclein levels were normalized to β-actin levels, a housekeeping gene. Graphed data are mean ± SEM. Significant differences from values of control mice by unpaired Student’s *t*-test are indicated as *P < 0.05; **P < 0.01; ***P < 0.001. CON, control; MPTP/p, MPTP and probenecid.

**FIGURE 7:**
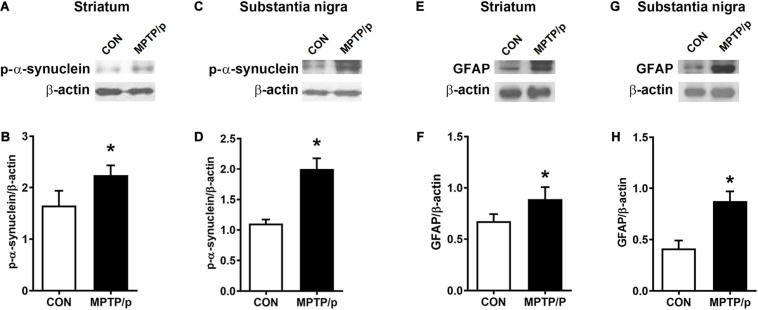
The chronic injection of MPTP/p leads to high expression levels of p-α-synuclein and GFAP in mice. Representative images of Western blotting analysis of p-α-synuclein in panels **(A)** striatum and **(C)** substantia nigra (*n* = 4/group). Representative images of Western blotting analysis of GFAP in panels **(E)** striatum and **(G)** substantia nigra (*n* = 4/group). The other three Western blot images are shown in Supplementary Figure 3. **(B,D,F,H)** Quantification of normalized p-α-synuclein levels and GFAP levels in each region. The p-α-synuclein levels and GFAP levels were normalized to β-actin levels, a housekeeping gene. Graphed data are mean ± SEM. Significant differences from values of control mice by unpaired Student’s *t*-test are indicated as *P < 0.05. CON, control; MPTP/p, MPTP and probenecid; p-α-synuclein, phosphorylated-α-synuclein; GFAP, glial fibrillary acidic protein.

α-Synuclein is a major pathological protein underlying PD pathogenesis (Xu and Pu, 2016). Most non-motor dysfunctions in PD are associated with α-synuclein pathology as well as the dopaminergic striatonigral system (Jellinger, 2011). Thus, we examined α-synuclein levels in the striatum and substantia nigra of the MPTP/p-treated mice. As shown in Figures 6A–D and Supplementary Figure 2, the MPTP/p injection showed significant increases in expression levels of α-synuclein in the striatum (*P* = 0.0005) and substantia nigra (*P* = 0.0053). We further identified the abnormal accumulation of α-synuclein in regions associated with non-motor symptoms, i.e., olfactory bulb (Henrich et al., 2018), hippocampus (Flores-Cuadrado et al., 2016), amygdala (Henrich et al., 2018), prefrontal cortex (Stoyka et al., 2020), locus coeruleus (Butkovich et al., 2018) and colon (Shannon et al., 2012). The results of Western blot at these areas showed a similar trend to the expression levels of α-synuclein of the striatum and substantia nigra. The expression levels of α-synuclein were significantly enhanced in the olfactory bulb (*P* = 0.0375), hippocampus (*P* = 0.0012), amygdala (*P* = 0.0308), prefrontal cortex (*P* < 0.0001), locus coeruleus (*P* = 0.0174) and colon (*P* = 0.0027) of the MPTP/p-treated mice (Figures 6E–P and Supplementary Figure 2). In addition, the expression levels of p-α-synuclein were significantly increased in the striatum (*P* = 0.0127), substantia nigra (*P* = 0.0260), olfactory bulb (*P* = 0.0294), hippocampus (*P* = 0.0273), amygdala (*P* = 0.0043) and locus coeruleus (*P* = 0.0133) (Figures 7A–D and Supplementary Figures 3A,B, 4A–F,I,J). Interestingly, the expression levels of GFAP were also significantly increased in the striatum (*P* = 0.0320) and substantia nigra (*P* = 0.0322) (Figures 7E–H and Supplementary Figures 3C,D). However, the expression levels of p-α-synuclein in the prefrontal cortex and colon (Supplementary Figures 4G,H,K,L) and the those of Iba-1 in the striatum and substantia nigra (Supplementary Figure 5) showed the trends of increase in the MPTP/p group, but they did not reach statistically significant.

### The Chronic Injection of MPTP/p Leads to Dopaminergic Neurons Loss in Mice

**FIGURE 8:**
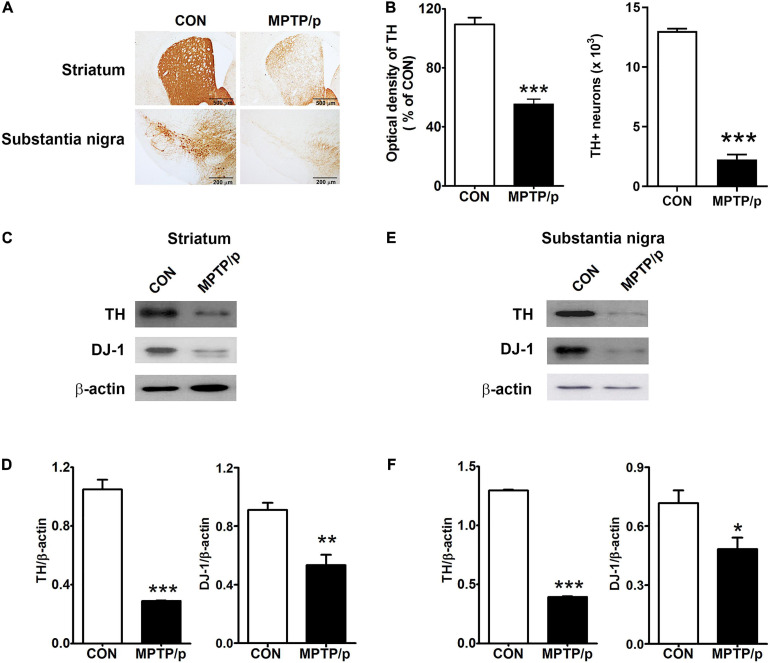
The chronic injection of MPTP/p leads to dopaminergic neurons loss in mice. Microphotographs of **(A, upper)** TH + dopaminergic fibers in striatum and **(A, lower)** TH + dopaminergic neurons in substantia nigra by TH immunostaining (*n* = 4/group). **(B, left panel)** The optical density of TH + dopaminergic fibers in striatum and **(B, right panel)** stereology counts of TH + dopaminergic neurons in substantia nigra. Unbiased stereological counting was conducted in substantia nigra. Representative images of Western blotting analysis using anti-TH and anti-DJ-1 antibodies in panels **(C)** striatum and **(E)** substantia nigra (*n* = 4/group). The other three Western blot images are shown in Supplementary Figure 6. Quantification of normalized TH and DJ-1 levels in panels **(D)** striatum and **(F)** substantia nigra. TH and DJ-1 levels were normalized to β-actin levels, a housekeeping gene. Graphed data are mean ± SEM. Significant differences from values of control mice by unpaired Student’s *t*-test are indicated as *P < 0.05; **P < 0.01; ***P < 0.001. CON, control; MPTP/p, MPTP and probenecid; TH, tyrosine hydroxylase.

Finally, we confirmed the dopaminergic depletion in the MPTP/p-treated mice. TH which is a critical enzyme for dopamine biosynthesis, is commonly used to detect dopaminergic neurons (Ishikawa et al., 2010). TH is a transcriptional target gene for DJ-1 which is a neuroprotective transcriptional activator (Zhong et al., 2006; Ishikawa et al., 2010). Thus, we clarified whether the non-motor and motor impairments in the MPTP/p-treated mice would result from dopaminergic neurons loss by analyzing TH and DJ-1 levels. The MPTP/p-induced dopaminergic depletion was examined by staining TH in the striatum and substantia nigra. TH + neurons counts were monitored by unbiased stereologic methods. As shown in Figures 8A,B the significant losses of both TH + dopaminergic fibers (*P* < 0.001) in the striatum and TH + neurons (*P* < 0.0001) in the substantia nigra were observed in the MPTP/p-treated mice, consistently with the non-motor and motor defective behaviors. The quantified TH expression levels by Western blot analysis were reduced in the striatum (*P* < 0.001) and substantia nigra (*P* < 0.001) of the MPTP/p-treated mice (Figures 8C–F and Supplementary Figure 6). Also, DJ-1 expression levels were significantly decreased in the striatum (*P* = 0.0050) and substantia nigra (*P* = 0.0366) of the MPTP/p-treated mice (Figures 8C–F and Supplementary Figure 6), indicating a similar vulnerability of the dopamine neurons observed in PD. Interestingly, DJ-1 expression level was reduced in the olfactory bulb of the MPTP/p-treated mice (Supplementary Figure 7, *P* < 0.0001).

## Discussion

In the current study, we determined that the chronic injection of MPTP/p caused olfactory deficit by performing the buried food test and short-term olfactory memory test; thermal hyperalgesia by the hot plate test; neuropsychiatric features by the open field test and tail suspension test; cognitive decline by the Y maze test and novel object recognition test; gastrointestinal dysfunction by the bead expulsion test and one-h stool collection test. Moreover, we confirmed that the MPTP/p-treated mice exhibited motor dysfunctions in the rotarod test, rearing test, catalepsy test, and akinesia test. The chronic injection of MPTP/p resulted in high expression levels of α-synuclein in striatum, substantia nigra, olfactory bulb, hippocampus, amygdala, prefrontal cortex, locus coeruleus, and colon, and low expression levels of TH in striatum and substantia nigra. Therefore, the current study established the experimental model of PD representing the comprehensive non-motor symptoms through the abnormal accumulation of α-synuclein and loss of dopaminergic neurons by the chronic injection of MPTP/p.

In PD, the causes of non-motor symptoms are multifactorial and are not described by a single lesion (Jellinger, 2011). The majority of PD patients had more than one non-motor dysfunction and the increased comorbidity of non-motor dysfunctions, which was associated with the severity of PD (Shulman et al., 2001). Autonomic symptoms, anxiety, cognitive decline, dementia, and psychotic episodes were all related to a high risk of depression in PD patients (Santangelo et al., 2014). Gastrointestinal dysfunctions in PD patients were associated with the deterioration of mobility, cognition and communication (Tibar et al., 2018). Although the MPTP-induced mouse models of PD faithfully have reproduced the naturally occurring neurodegeneration, following similar topographical patterns of human PD (Meredith and Rademacher, 2011), the comprehensive alterations in the non-motor functions using the MPTP-treated mice have not been noticed in any one study (Schapira et al., 2017). Dopaminergic neurons died quickly in acute and subchronic MPTP models, whereas the chronic MPTP model including MPTP/p showed to be more progressive loss of dopaminergic neurons (Meredith and Rademacher, 2011). Thus, in the current study, we injected MPTP/p for 5 weeks and established the experimental chronic mouse model of PD mimicking the comprehensive non-motor dysfunctions of human PD, showing olfactory deficit, thermal hyperalgesia, anxiety, depression, cognitive decline, and gastrointestinal dysfunction by conducting various behavior tests. We clearly characterized the mouse model of PD, fulfilling many of the main criteria of human conditions including non-motor and motor impairments. Although previous studies have reported non-motor symptoms by conducting the buried food test (Choi et al., 2018), open field test (Choi et al., 2018) and bead expulsion test (Choi et al., 2018) in the chronic MPTP/p-treated mice, we conducted the short-term olfactory memory test, hot plate test, tail suspension test, Y maze test, novel object recognition test and one-h stool collection test in the chronic MPTP/p-treated mice for the first time and established the chronic mouse model of PD mimicking the comprehensive non-motor symptoms of human PD. Thus, the current study is noteworthy in that conducting the various behavior tests in one model is a more advanced approach to the symptomatic study in PD patients. However, as animal models using toxins such as 6-OHDA or rotenone have previously been demonstrated to be useful for studying non-motor dysfunctions of human PD (Blesa and Przedborski, 2014), further investigation is needed to characterize the comprehensive non-motor symptoms in various chronic models of PD. The current PD model has been established using male mice with reference to previous reports that the incidence rate of PD in men was higher than that in women (Van Den Eeden et al., 2003; Miller and Cronin-Golomb, 2010). However, further studies are needed in female mice to clarify the current model. Further researches are also required to develop this model system into a validated model as a clinical research platform.

A characteristic neuropathological hallmark of PD is Lewy bodies which consists predominantly of α-synuclein (Spillantini et al., 1997). The appearance of α-synuclein in a series of brain regions coincides with non-motor and motor dysfunctions in PD patients (Gajula Balija et al., 2011; Schirinzi et al., 2019). Hyposmia is implicated in olfactory bulb and amygdala; pain in locus coeruleus containing Lewy bodies and amygdala; anxiety or depression in locus coeruleus; cognitive dysfunction in frontal cortex and hippocampus (Jellinger, 2011; Schapira et al., 2017). In particular, the accumulation of α-synuclein in substantia nigra, olfactory bulb, hippocampus, amygdala, prefrontal cortex, and locus coeruleus replicated non-motor features of human PD pathology such as anxiety or cognitive dysfunction (Flores-Cuadrado et al., 2016; Schapira et al., 2017; Butkovich et al., 2018; Henrich et al., 2018; Stoyka et al., 2020). The accumulation of α-synuclein was also found in colon of PD patients, which innervated the intestine, promoted the progression of α-synuclein pathology to the brain, caused behavioral dysfunction and allowed early detection of PD before the slow onset of motor symptoms (Schapira et al., 2017; Challis et al., 2020). The current study showed the high expression levels of α-synuclein in olfactory bulb, hippocampus, amygdala, prefrontal cortex, locus coeruleus, and colon with the nigrostriatal areas of the MPTP/p-treated mice, which was consistent with previous studies describing that the mice overexpressing α-synuclein in midbrain exhibited the non-motor impairments with progressive motor decline (Nuber et al., 2008; Schapira et al., 2017). The p-α-synuclein, pS129, level was higher in PD patients than healthy controls (Stewart et al., 2015). Our results showed that the expression levels of pS129 were increased in the striatum and substantia nigra of the MPTP/p-treated mice. Thus, the current study suggests that the chronic injection of MPTP/p would reproduce behavioral phenotypes of human PD through the abnormal distribution of α-synuclein in various brain regions and colon. Recently, Kim et al. (2019) established a gastrointestinal α-synuclein-injected PD mouse model characterized by PD with both motor and non-motor symptoms, suggesting it as a new model with α-synucleinopathies, one of the main features of PD. Kim et al. (2019) conducted a variety of behavioral tests and demonstrated that the PD mouse model had several non-motor symptoms, but a significant loss of dopaminergic neurons in substantia nigra and dysfunction of motor and non-motor symptoms were observed at 7 months after α-synuclein injection. Our chronic MPTP/p-injected model has shown that the loss of dopaminergic neurons, the distribution of α-synuclein and comprehensive non-motor symptoms are implicated within a short period of time compared to the previous study of Kim et al. (2019). Thus, we suggest that the current PD mouse model by MPTP/p would be suitable for short-term pharmacological and pathological studies, while the study of Kim et al. (2019) would be suitable for long-term studies by α-synuclein on gut-brain axis in PD.

PD is characterized by motor deficits via a slow and progressive loss of nigral dopaminergic neurons (Maiti et al., 2017). The dopaminergic neurodegeneration of PD is also involved in the genesis of non-motor symptoms (Lee and Koh, 2015). Dopamine is synthesized by l-DOPA converted by TH and l-DOPA decarboxylase (Daubner et al., 2011). DJ-1 has a neuroprotective activity against neurodegeneration in PD and loss of DJ-1 function triggers the onset of PD (Ariga et al., 2013). Our results have revealed the chronic injection of MPTP/p dramatically leads to low expression levels of TH and DJ-1 in striatum and substantia nigra, and the losses of both TH + dopaminergic fibers in striatum and TH + neurons in substantia nigra, which was consistent with previous studies determining chronic effects of MPTP/p injection (Biju et al., 2018; Shao et al., 2019). Astrocytes are associated with the progression of PD by the production of pro-inflammatory cytokines which damage dopaminergic neurons (Rappold and Tieu, 2010). The overexpression of GFAP often occur in PD patients and these alterations of GFAP in astrocytes are involved in the pathogenesis of PD (Clairembault et al., 2014). Emamzadeh and Surguchov (2018) suggested that PD can be identified with high levels of GFAP. The current study showed the elevated levels of GFAP in the striatum and substantia nigra. Thus, we suggest that this model would be excellently suited to study the non-motor dysfunctions of PD by loss of dopaminergic nigrostriatal neurons with high levels of GFAP. However, various proteins, as well as TH and α-synuclein, are involved in the mechanisms underlying pathological and behavioral phenotypes of PD (Blesa and Przedborski, 2014). Whether the presence of various pathologic proteins contributes to these non-motor symptoms of PD will require further study.

Nutrition plays a critical role in both neuroprotection and neurodegeneration of PD (Seidl et al., 2014). The MPTP injection can cause dystrophy in mice, which can lead to weight loss (Sedelis et al., 2001). On the last day of MPTP/p injection, there was a significant difference in body weight between control group and MPTP/p group (Supplementary Figure 8, *P* < 0.0001). Although not fully pathologically understood in the current study, body weight loss and nutrition status may have an effect on behavioral tests. Thus, future research is needed to identify the association between body weight and behavioral tests in the current model. Regardless of the limitations, the current study suggests the chronic injection of MPTP/p would reproduce behavioral phenotypes of human PD through the abnormal accumulation of α-synuclein and loss of dopaminergic neurons.

In summary, the current study shows that the chronic intraperitoneal injection of MPTP/p leads to the abnormal accumulation of α-synuclein in a series of brain regions and colon and the degeneration of dopaminergic neurons in the striatum and substantia nigra, which is involved in various non-motor symptoms. Because attention is now focused on non-motor dysfunctions to improve the quality of life of PD patients and the comprehensive focus of non-motor aspects in the experimental model of PD is an evolution from previous studies on the non-motor aspects, the current study will provide significant insights into the various pathological properties of PD. However, further studies are required to clarify the non-motor symptoms of PD pathogenesis through multiple signaling pathways in the current chronic MPTP/p-induced PD model.

## Data Availability Statement

The raw data supporting the conclusions of this article will be made available by the authors, without undue reservation.

## Ethics Statement

Experimental procedures were performed with reference to the National Institutes of Health Guide for the Care and Use of Laboratory Animals. The procedures were also approved by the Dongguk University Animal Care Committee (IACUC-2017-023-1).

## Author Contributions

HL and H-JP supervised the study and reviewed the manuscript. N-RH, Y-KK, SA, T-YH, HL, and H-JP conducted the experiments and data analysis. N-RH wrote the manuscript. All authors contributed to the article and approved the submitted version.

## Conflict of Interest

The authors declare that the research was conducted in the absence of any commercial or financial relationships that could be construed as a potential conflict of interest.
