# Supplementary material for: A Comprehensive Phenotype of Non-motor Impairments and Distribution of Alpha-Synuclein Deposition in Parkinsonism-Induced Mice by a Combination Injection of MPTP and Probenecid
Source: Front Aging Neurosci. 2021 Jan 13;12:599045. doi: 10.3389/fnagi.2020.599045 (PMC7838388; doi:10.3389/fnagi.2020.599045)
Supplement: Supplementary file 1 [file Data_Sheet_1.docx]

Supplementary Material

# Supplementary Figures


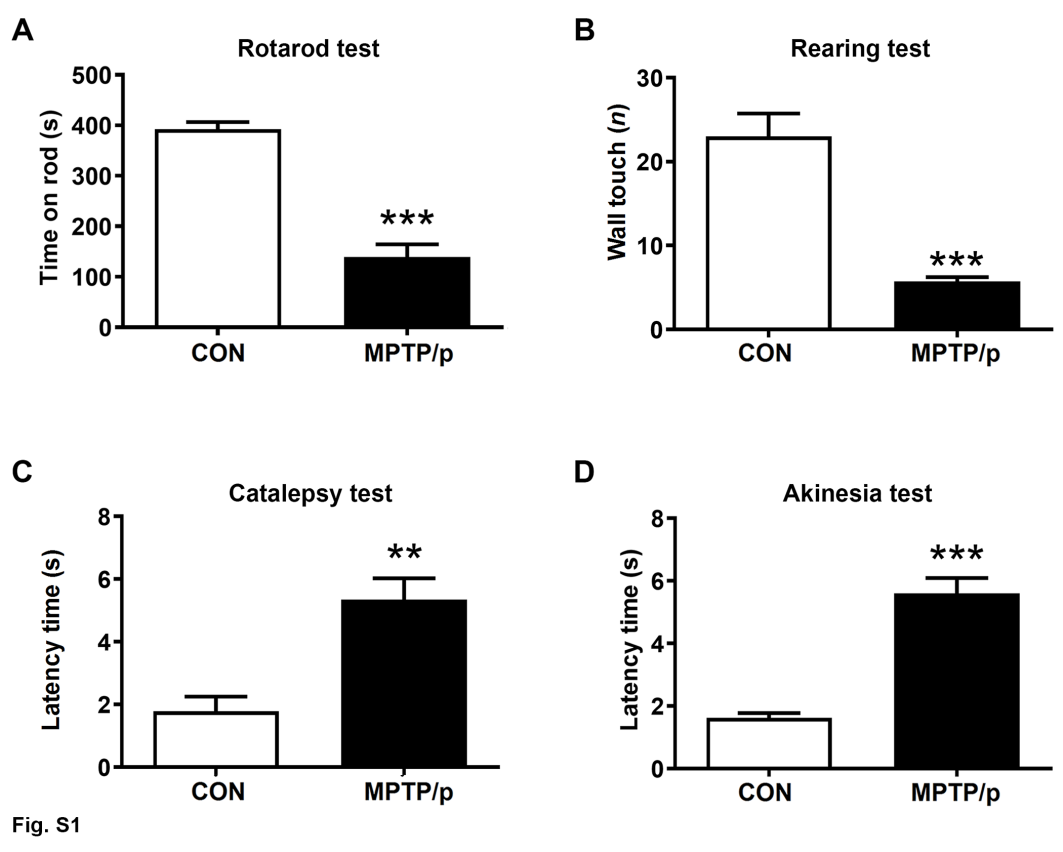


**Supplementary Figure S1.** The chronic injection of MPTP/p disrupts motor function in mice. MPTP/p was intraperitoneally administered twice a week for 5 weeks, a total of 10 times. Behavioral measurements on the MPTP/p-treated mice were as follows: **(A)** time (s) on rod in the rotarod test (*n* = 14/group) on day 35 of exposure to MPTP/p, **(B)** the number (*n*) of wall touch in the rearing test (*n* = 7/group) on day 36 of exposure to MPTP/p, **(C)** latency time (s) in the catalepsy test (*n* = 7/group) on day 36 of exposure to MPTP/p, **(D)** latency time (s) in the akinesia test (*n* = 14/group) on day 40 of exposure to MPTP/p described in Materials and methods section. Graphed data are mean ± SEM. Significant differences from values of control mice by unpaired Student’s *t*-test are indicated as ^**^*P* < 0.01; ^***^*P* < 0.001. CON, control; MPTP/p, MPTP and probenecid.


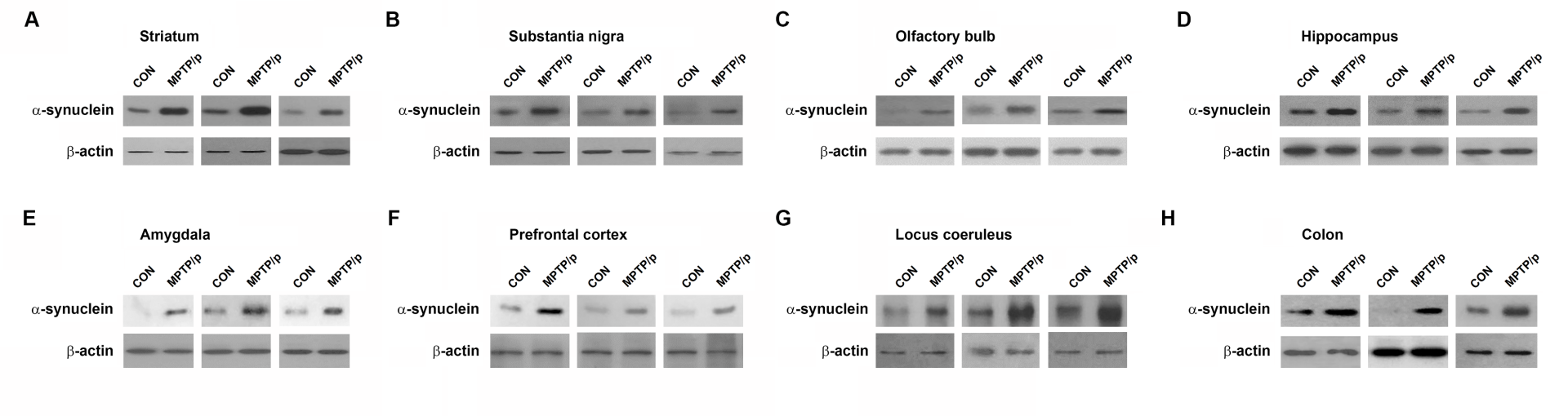


**Supplementary Figure S2.** Membrane images of Western blots for Figure 6 (n = 4/group). CON, control; MPTP/p, MPTP and probenecid.


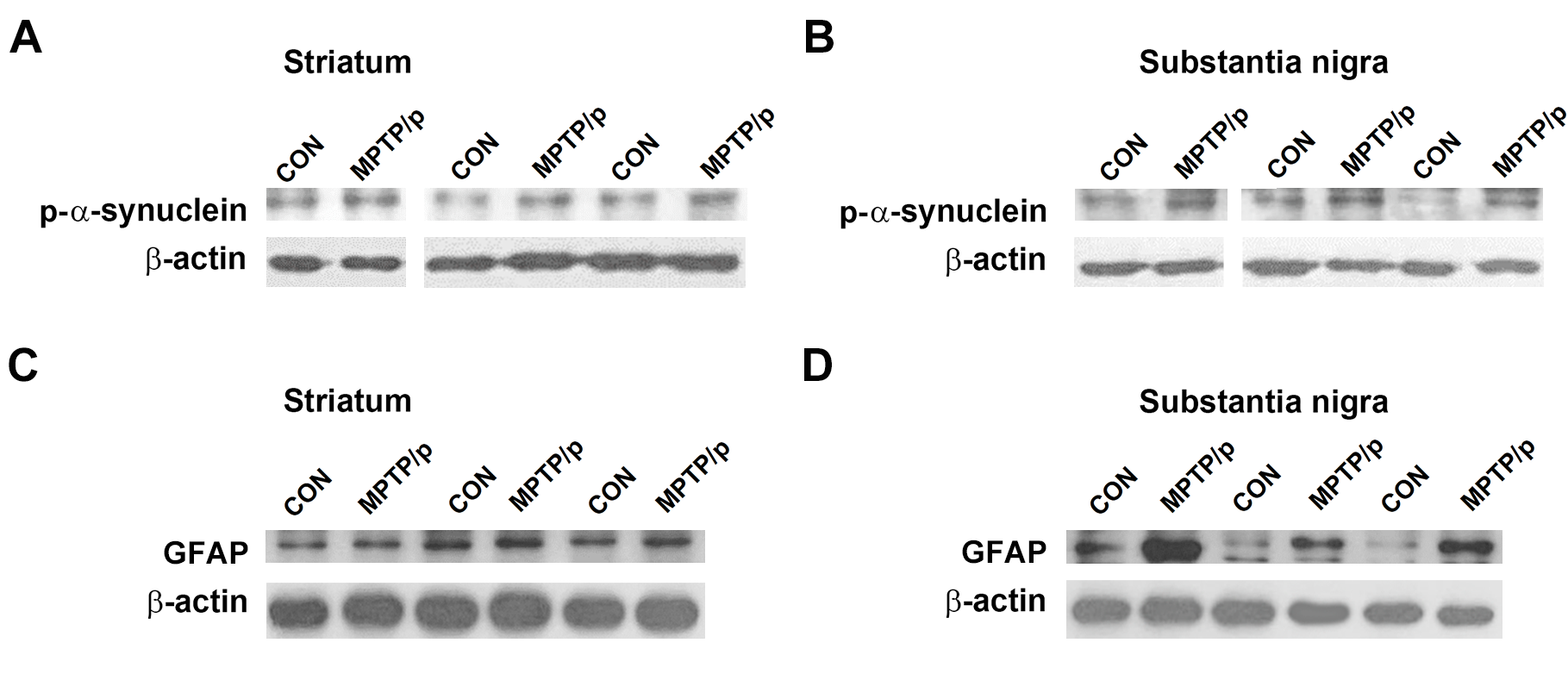


**Supplementary Figure S3.** Membrane images of Western blots for Figure 7 (n = 4/group). CON, control; MPTP/p, MPTP and probenecid; p-α-synuclein, phosphorylated-α-synuclein; GFAP, glial fibrillary acidic protein.


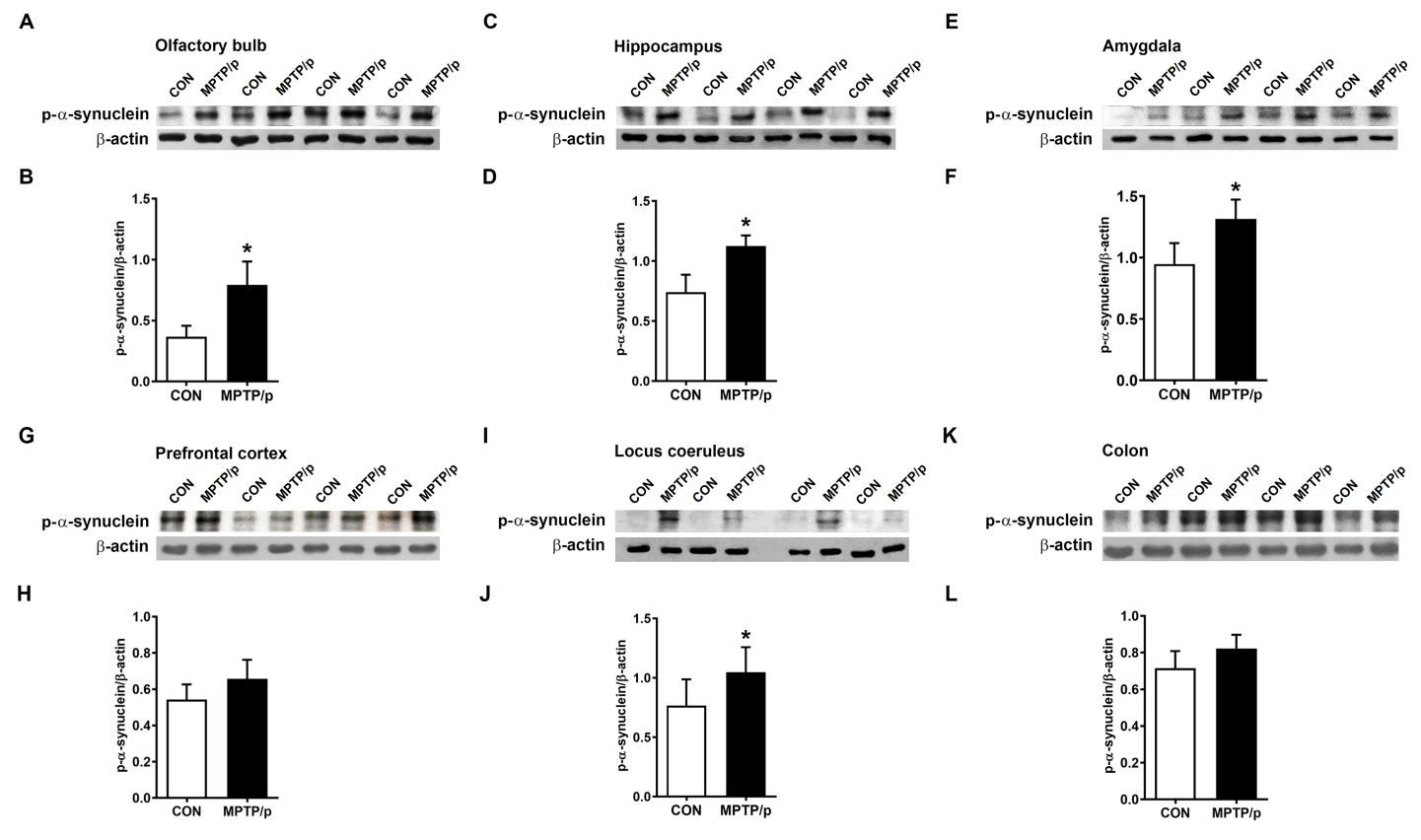


**Supplementary Figure S4.** Membrane images of Western blots using an anti-p-α-synuclein antibody in **(A)** olfactory bulb, **(C)** hippocampus, **(E)** amygdala, **(G)** prefrontal cortex, **(I)** locus coeruleus and **(K)** colon (*n* = 4/group). **(B,D,F,H,J,L)** Quantification of normalized p-α-synuclein levels. The p-α-synuclein levels were normalized to β-actin levels, a housekeeping gene. Graphed data are mean ± SEM. CON, control; MPTP/p, MPTP and probenecid; p-α-synuclein, phosphorylated-α-synuclein.


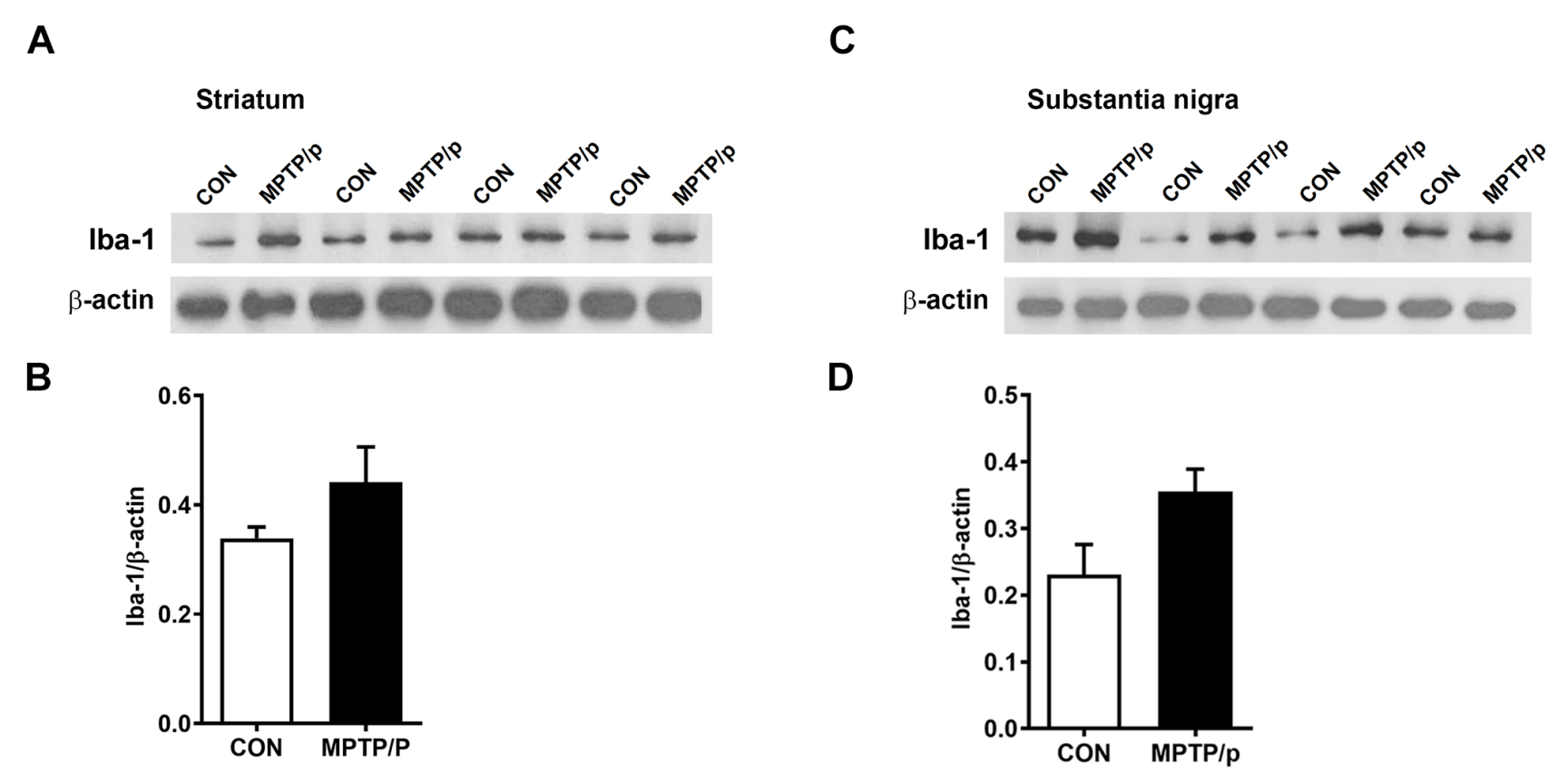


**Supplementary Figure S5.** Membrane images of Western blots using an anti-Iba-1 antibody in **(A)** striatum and **(C)** substantia nigra (*n* = 4/group). **(B,D)** Quantification of normalized Iba-1 levels. Iba-1 levels were normalized to β-actin levels, a housekeeping gene. Graphed data are mean ± SEM. CON, control; MPTP/p, MPTP and probenecid.


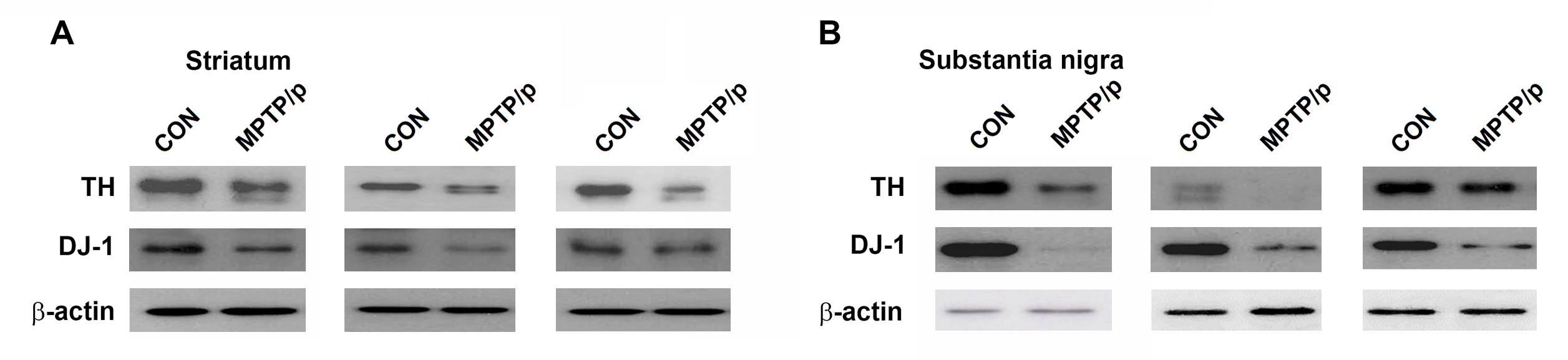


**Supplementary Figure S6.** Membrane images of Western blots for Figure 8C-F (n = 4/group). CON, control; MPTP/p, MPTP and probenecid; TH, tyrosine hydroxylase.


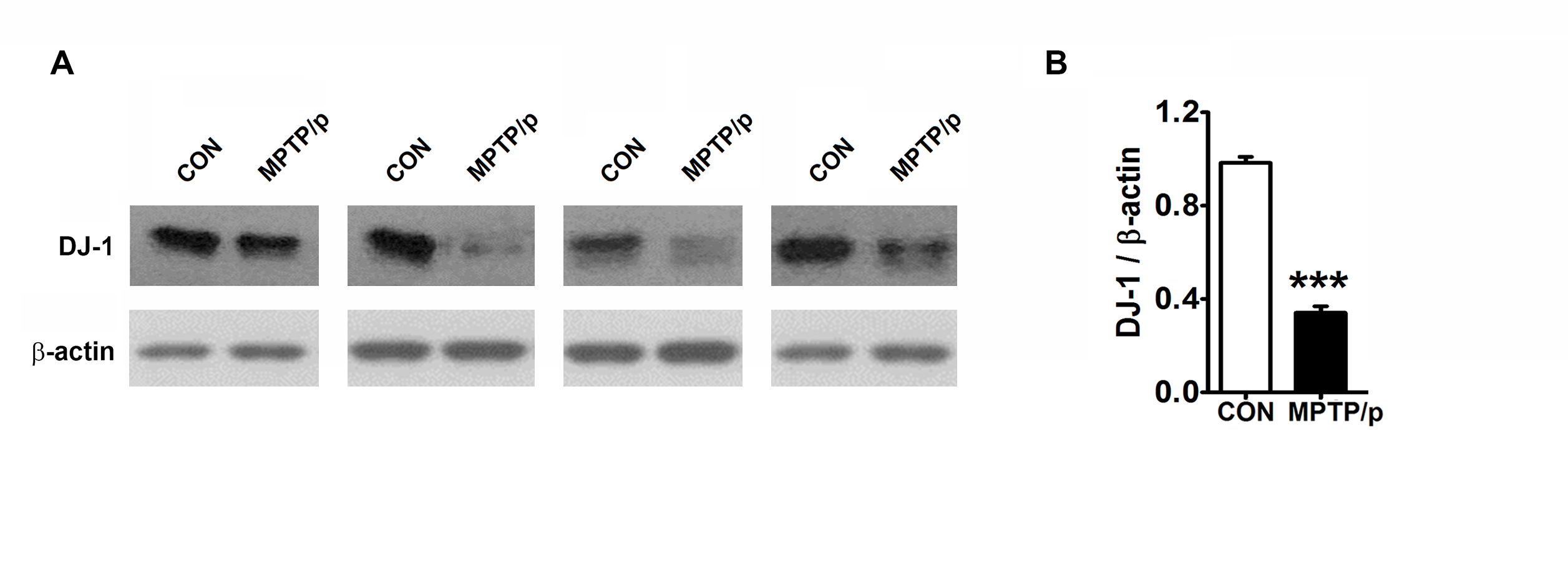


**Supplementary Figure S7. (A)** Membrane images of Western blots using an anti-DJ-1 antibody in olfactory bulb (*n* = 4/group). **(B)** Quantification of normalized DJ-1 levels. DJ-1 levels were normalized to β-actin levels, a housekeeping gene. Graphed data are mean ± SEM. Significant differences from values of control mice by unpaired Student’s *t*-test are indicated as ^***^*P <* 0.001. CON, control; MPTP/p, MPTP and probenecid.


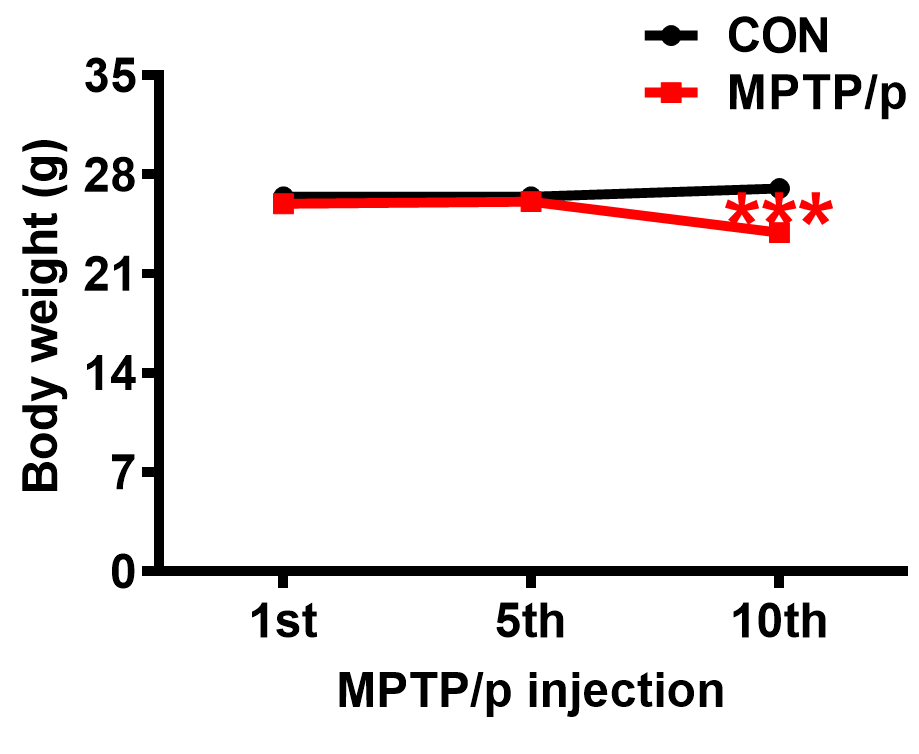


**Supplementary Figure S8.** Body weight measurements at the first, 5th and 10th injections of MPTP/p. Graphed data are mean ± SEM. Significant differences from values of control mice by unpaired Student’s *t*-test are indicated as ^***^*P <* 0.001. CON, control; MPTP/p, MPTP and probenecid.
